# Bias Associated with Mining Electronic Health Records

**DOI:** 10.5210/disco.v6i0.3581

**Published:** 2011-06-06

**Authors:** George Hripcsak, Charles Knirsch, Li Zhou, Adam Wilcox, Genevieve B Melton

**Affiliations:** 1 Department of Biomedical Informatics, Columbia University, New York, NYUnited States; 2 Pfizer Inc., New York, NYUnited States; 3 Partners Healthcare, Boston, MAUnited States; 4 Department of Surgery, University of Minnesota, Minneapolis, MNUnited States

## Abstract

Large‐scale electronic health record research introduces biases compared to traditional manually curated retrospective research. We used data from a community‐acquired pneumonia study for which we had a gold standard to illustrate such biases. The challenges include data inaccuracy, incompleteness, and complexity, and they can produce in distorted results. We found that a naïve approach approximated the gold standard, but errors on a minority of cases shifted mortality substantially. Manual review revealed errors in both selecting and characterizing the cohort, and narrowing the cohort improved the result. Nevertheless, a significantly narrowed cohort might contain its own biases that would be difficult to estimate.

## Background

With the increasing adoption of electronic health records, there is the potential for over one billion patient visits to be documented per year in the US [1], and these data should be a boon to clinical research [2]. Large-scale electronic health record-based research is more challenging than traditional retrospective studies, however. The record is frequently inaccurate [3], incomplete, and complex. In traditional retrospective research, a human expert reads the data sources—which may include electronic health records—for each subject, interprets them, and records more reliable variables. Data that are obviously inaccurate or contradictory are adjudicated, missing variables are frequently filled in by inferring related information from other variables, and deeply nested information is interpreted in the context of the study.

Large-scale electronic health record-based research hopes to process huge numbers of subjects without subject-by-subject human intervention. To duplicate retrospective research, study analysts must attempt to mimic the reasoning that researchers apply to individual records. In this paper, we illustrate the challenge.

In 1997, Fine et al. [4] used data from the Pneumonia Patient Outcomes Research Team cohort study to define a pneumonia severity index that could help clinicians decide whether a patient with community-acquired pneumonia needed to be admitted for treatment or could be discharged home on oral antibiotics. The index used five severity classes from I (healthiest) to V (most severe) and reported the mortality rate for each class. The 1997 study is an example of traditional retrospective research.

In 2007, Hripcsak et al. [5] used the New York-Presbyterian Hospital clinical data warehouse to repeat Fine’s study. The informatics research goal was to demonstrate the use of discordance in a subsample to improve data mining. The 2007 study is an example of large-scale electronic health record research.

In this paper, we reuse the data from the 2007 study to illustrate the biases inherent in a naïve use of electronic health record data, graphing the data to emphasize the biases. We use the 1997 Fine study to serve as the gold standard.

## Methods 

The Fine classification uses a broad range of clinical information, including demographics (age, gender, nursing home residence), coexisting illnesses (neoplastic, liver, heart failure, cerebrovascular, renal), physical findings (mental status, respiratory rate, blood pressure, temperature, pulse), and laboratory findings (pH, BUN, sodium, glucose, hematocrit, PO2, pleural effusion). Class I patients are those under 50 years old with no coexisting illnesses or physical findings. Other patients are assigned a numeric score based on their findings, and the score is mapped to classes II to V.

Repeating the Fine study using the electronic health record required two steps: (1) defining a cohort of patients with community-acquired pneumonia, and (2) calculating the Fine score.  The required variables for both steps were derived from the New York-Presbyterian Hospital electronic health record’s administrative data including ICD9-CM codes, laboratory data, radiology data, and clinical notes whose symptoms, physical findings and diagnoses were coded via natural language processing.

The community-acquired pneumonia cohort was defined as patients who had some indication of pneumonia—a relevant ICD9-CM code, mention of pneumonia in a discharge summary, or pneumonia-relevant findings in a chest radiographic report—corroborated by radiographic evidence of pneumonia and at least one symptom or sign consistent with pneumonia. A case was considered community acquired if there was no evidence of HIV and there were no recent episodes of pneumonia or hospitalization. The Fine classification was then applied to the case, and a mortality rate and a 95% confidence interval was calculated for each class.

The institutional review board approved the study.

## Results

**Figure 1.**
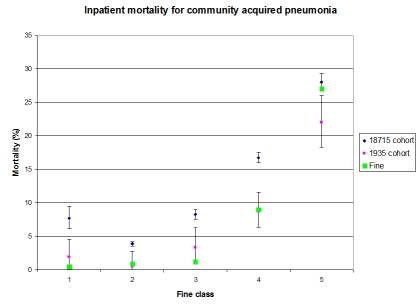
Inpatient mortality for community acquired pneumonia.

Between the years 1996 and 1999, the clinical data warehouse had 49,642 inpatient and ambulatory cases that had some indication of pneumonia, and 18,715 cases had corroboratory evidence and were considered community acquired. Figure 1 shows Fine’s mortality results [52] for each severity class as large green squares. The blue diamonds with error bars show the calculated classes and mortality for the 18,715-case cohort [5]. While the 18,715-case cohort roughly follows the Fine results, every error bar excludes the corresponding Fine result, and Class I (i.e., the healthiest) showed almost twice the mortality of Class II.

A manual review revealed two main problems: (1) many subjects did not actually have pneumonia and (2) many subjects in Class I should have been in Class III or higher, including several intensive care unit cases. Pneumonia findings were not specific enough, allowing patients with cardiac arrest, heart and lung transplant, sepsis, respiratory distress syndrome, cystic fibrosis, and dissecting aortic aneurysm to enter the cohort without having pneumonia. Furthermore, important findings were insufficiently documented, such as liver failure, myocardial infarction, congestive heart failure, and HIV. If a patient came in with severe community-acquired pneumonia and died in the emergency department, then the care team spent little time documenting symptoms so that in the electronic health record, the patient appeared to be healthy other than the death.

We then narrowed the cohort by requiring an ICD9-CM diagnosis of pneumonia (and not mere sepsis, as others have defined it) in addition to the other NLP-related criteria and by requiring that a discharge summary be available for inpatients. Because very short admissions did not require discharge summaries, patients who arrived and died immediately were filtered out. Using the additional criteria reduced the sample to 1935 cases (or about 10% of the previous sample). The results are shown in Figure 1 as small pink circles with error bars [5]. The new results track the Fine results, and after correcting for multiple hypotheses, the two samples are not statistically different. The nominal mortality for Class I is still slightly higher than that for Class II, but confidence intervals overlap and they are much closer than before. The mortality in Class V is nominally lower than Fine’s result, and this may be due to inadvertent filtering of the most severe cases.

## Discussion

This study reveals several lessons about large-scale electronic health record research:

1. The electronic health record does contain useful information. Even the naïve approach roughly matched the gold standard, and the filtered cohort matched it closely. Manual review revealed that the majority of cases were correctly classified.

2. Errors can occur both in selecting a cohort and in characterizing that cohort. Both types of errors occurred in this study.

3. Errors in even a small number of cases can have a relatively large effect on the outcome. For example, in this study, the small number of patients who died in the emergency department had a large effect on the estimated mortality.

4. Adding simple constraints to narrow the cohort produced better results, but it eliminated most of the cohort, opening an opportunity for bias. Had a gold standard not been available, it would have been difficult to tell whether the results were trustworthy.

5. Manual review of the cases or a sample of cases is invaluable in improving the sample. Most useful is reviewing cases at the “edges,” either at the extremes (e.g., the most and least severe cases in this study) or where they have the greatest leverage on the outcome (e.g., the deaths in this study).

We believe that more informatics research is needed to improve mining of electronic health records. Improving the accuracy and completeness of the record will clearly be valuable. Other avenues include finding ways to estimate bias, identifying cases most likely to need reviewing [5], and triangulating from redundant information in the record.

## Acknowledgments

This work was supported by the National Library of Medicine (R01 LM06910) “Discovering and applying knowledge in clinical databases” and by Pfizer, Inc.
